# Supplementary material for: Study of Camellia sinensis diploid and triploid leaf development mechanism based on transcriptome and leaf characteristics
Source: PLoS One. 2023 Feb 17;18(2):e0275652. doi: 10.1371/journal.pone.0275652 (PMC9937487; doi:10.1371/journal.pone.0275652)
Supplement: Attachment — Submitted filename: Comments.pdf [file pone.0275652.s004.pdf]

Comments to the author (if any):
Reviewer #1: General comment
This manuscript studied functional traits and photosynthetic characteristics of diploid and triploid tea leaves, and investigated the molecular mechanisms through transcriptome analysis.
The subject of this study is my interest. The study is well documented and designed. Findings and data are important.

There are only minor points to review.

Results
Line 199: It was mentioned that 'the stomata density of triploid leaves was significantly lower than that of diploids; the stomata density of diploid tea leaves was twice that of triploid stomata'. But it was not explained well in discussion why the stomatal density is higher in diploids. It must be clarified.

Figures
The resolution of Figure 4 and 6 are not good and legible.
