# Supplementary material for: Study of Camellia sinensis diploid and triploid leaf development mechanism based on transcriptome and leaf characteristics
Source: PLoS One. 2023 Feb 17;18(2):e0275652. doi: 10.1371/journal.pone.0275652 (PMC9937487; doi:10.1371/journal.pone.0275652)
Supplement: Attachment — Submitted filename: Response.docx [file pone.0275652.s005.docx]

Journal Requirements:

When submitting your revision, we need you to address these additional requirements.

1. Please ensure that your manuscript meets PLOS ONE's style requirements, including those for file naming. The PLOS ONE style templates can be found at
   [https://journals.plos.org/plosone/s/file?id=wjVg/PLOSOne_formatting_sample_main_body.pdf](https://journals.plos.org/plosone/s/file?id=wjVg/PLOSOne_formatting_sample_main_body.pdf) and
   [https://journals.plos.org/plosone/s/file?id=ba62/PLOSOne_formatting_sample_title_authors_affiliations.pdf](https://journals.plos.org/plosone/s/file?id=ba62/PLOSOne_formatting_sample_title_authors_affiliations.pdf)

Response:The manuscript has been revised in the style of PLOSE ONE

2. We noticed your current submission has significant text overlap with the following preprint:
[https://www.researchsquare.com/article/rs-20102/v2](https://www.researchsquare.com/article/rs-20102/v2)

Please specify the relation and difference between the two studies. If they are the same study, please specify the reasons for the authorship change.

Response:The two studies are basically the same. The main reasons: 1) When submitting the BMC Plant biology journal, I accidentally selected the preprint version, and the manuscript was rejected, and the preprint version could not be cancelled. 2) We have made a lot of revisions and improvements to the manuscript, mainly completed by Yao Xinzhuan, Chen Hufang and Zhang Baohui, and the first author Qi Yong did not participate in the revision and improvement, so the order of authors changed.

3. We note that the grant information you provided in the ‘Funding Information’ and ‘Financial Disclosure’ sections do not match.

When you resubmit, please ensure that you provide the correct grant numbers for the awards you received for your study in the ‘Funding Information’ section.

Response:Thank you very much for your comments，Submission system modification.

4. Thank you for stating the following in your Competing Interests section:

"No"
 
Please complete your Competing Interests on the online submission form to state any Competing Interests. If you have no competing interests, please state ""The authors have declared that no competing interests exist."", as detailed online in our guide for authors at [http://journals.plos.org/plosone/s/submit-now](http://journals.plos.org/plosone/s/submit-now)

This information should be included in your cover letter; we will change the online submission form on your behalf.

Response: As required by the journal, it has been added to the recommendation letter.

5. In your Data Availability statement, you have not specified where the minimal data set underlying the results described in your manuscript can be found. PLOS defines a study's minimal data set as the underlying data used to reach the conclusions drawn in the manuscript and any additional data required to replicate the reported study findings in their entirety. All PLOS journals require that the minimal data set be made fully available. For more information about our data policy, please see [http://journals.plos.org/plosone/s/data-availability.](http://journals.plos.org/plosone/s/data-availability.)

Upon re-submitting your revised manuscript, please upload your study’s minimal underlying data set as either Supporting Information files or to a stable, public repository and include the relevant URLs, DOIs, or accession numbers within your revised cover letter. For a list of acceptable repositories, please see [http://journals.plos.org/plosone/s/data-availability#loc-recommended-repositories.](http://journals.plos.org/plosone/s/data-availability#loc-recommended-repositories.) Any potentially identifying patient information must be fully anonymized.

Important: If there are ethical or legal restrictions to sharing your data publicly, please explain these restrictions in detail. Please see our guidelines for more information on what we consider unacceptable restrictions to publicly sharing data: [http://journals.plos.org/plosone/s/data-availability#loc-unacceptable-data-access-restrictions.](http://journals.plos.org/plosone/s/data-availability#loc-unacceptable-data-access-restrictions.) Note that it is not acceptable for the authors to be the sole named individuals responsible for ensuring data access.

We will update your Data Availability statement to reflect the information you provide in your cover letter.

Response: Response: As required by the journal, it has been added to the recommendation letter.

6. PLOS requires an ORCID iD for the corresponding author in Editorial Manager on papers submitted after December 6th, 2016. Please ensure that you have an ORCID iD and that it is validated in Editorial Manager. To do this, go to ‘Update my Information’ (in the upper left-hand corner of the main menu), and click on the Fetch/Validate link next to the ORCID field. This will take you to the ORCID site and allow you to create a new iD or authenticate a pre-existing iD in Editorial Manager. Please see the following video for instructions on linking an ORCID iD to your Editorial Manager account: [https://www.youtube.com/watch?v=_xcclfuvtxQ](https://www.youtube.com/watch?v=_xcclfuvtxQ)

Response: As required by the journal, it has been added to the recommendation letter.

7. Please review your reference list to ensure that it is complete and correct. If you have cited papers that have been retracted, please include the rationale for doing so in the manuscript text, or remove these references and replace them with relevant current references. Any changes to the reference list should be mentioned in the rebuttal letter that accompanies your revised manuscript. If you need to cite a retracted article, indicate the article’s retracted status in the References list and also include a citation and full reference for the retraction notice.
Response: Revised in the manuscript as required by the Journal

Comments to the author (if any):

Reviewer #1: General comment

This manuscript studied functional traits and photosynthetic characteristics of diploid and

triploid tea leaves, and investigated the molecular mechanisms through transcriptome

analysis.

The subject of this study is my interest. The study is well documented and designed. Findings

and data are important.

There are only minor points to review.

Results

Line 199: It was mentioned that ′the stomata density of triploid leaves was significantly

lower than that of diploids; the stomata density of diploid tea leaves was twice that of

triploid stomata′. But it was not explained well in discussion why the stomatal density is

higher in diploids. It must be clarified.

Response:Thank you very much for your comments. In our manuscript, line 622-733, both explained that the stomatal density of triploid leaves was significantly.

Figures

The resolution of Figure 4 and 6 are not good and legible.

Response:Thank you very much for your comments. We have revised it in the manuscript.
